# Multiscale time-domain NMR for structural, water, and texture characterization of meat and plant-based food matrices: a mini review

**DOI:** 10.3389/fnut.2026.1770837

**Published:** 2026-03-13

**Authors:** Zeev Wiesman

**Affiliations:** Phyto-Lipid Biotech Lab (PLBL), Department of Biotechnology Engineering, Faculty of Engineering Sciences, Ben-Gurion University of the Negev, Be’er Sheva, Israel

**Keywords:** diffusion NMR, food matrices, meat systems, multiscale analysis, plant-based analogs, relaxometry, texture, time-domain NMR

## Abstract

Time-domain nuclear magnetic resonance (TD-NMR) is a powerful, non-destructive method for probing water mobility, molecular dynamics, and hierarchical structure in complex food matrices. Over the past several decades, numerous studies have demonstrated that relaxation, diffusion, and relaxometry-derived parameters are closely linked to microstructural constraints, water distribution, texture, and water-holding capacity (WHC) in both muscle and plant-based systems. This mini review synthesizes foundational and recent advances into a unified multiscale TD-NMR framework spanning molecular (μs–ms), mesoscopic (μm), and macroscopic (mm–cm) levels. We show how T₁/T₂ relaxation, diffusion coefficients, and multidimensional correlation methods can be mapped onto structural and functional properties of native meat and engineered plant-based matrices. Integrating these TD-NMR modalities with microstructural imaging and texture profiling provides a quantitative approach for mechanistic understanding and product optimization. Future opportunities include machine-learning tools for multidimensional datasets, physics-guided inversion algorithms, and the development of industrial, inline TD-NMR sensing platforms.

## Graphical abstract

**Figure:**
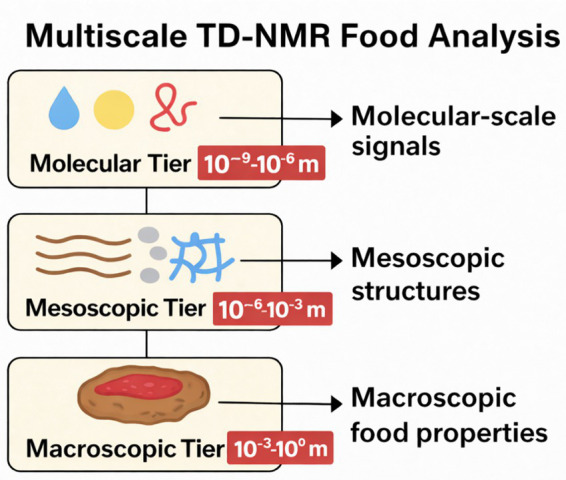
This diagram shows how TD-NMR links molecular mobility, mesoscopic microstructure, and macroscopic properties in food systems. Fast motions of water, proteins, and lipids shape relaxation and diffusion signals, which reflect pores, fibers, and protein networks. These multiscale interactions ultimately determine texture and water-holding capacity in meat and plant-based matrices.

## 1 Introduction

Understanding structure–function relationships in complex food matrices requires analytical methods capable of quantifying water dynamics, molecular mobility, and mesoscale architecture *in situ*. Time-domain nuclear magnetic resonance (TD-NMR) has emerged as a central technique due to its non-destructive nature, minimal sample preparation, and inherent sensitivity to hydration, microstructure, and phase mobility. TD-NMR measurements exploit the relaxation and diffusion behavior of nuclear spins—most commonly protons—following excitation by radiofrequency pulses (1–6).

Compared with other non-destructive analytical tools used in food structure analysis—such as near-infrared spectroscopy (NIR), hyperspectral imaging, ultrasound, or dielectric spectroscopy—TD-NMR offers a unique combination of sensitivity to *water mobility*, *molecular binding states*, and *microstructural confinement*. While optical and spectroscopic methods primarily provide compositional or surface-weighted information, TD-NMR directly probes hydrogen dynamics throughout the bulk matrix, enabling quantitative assessment of hydration topology, compartmentalization, and molecular mobility across multiple length scales. This intrinsic sensitivity to water–matrix interactions positions TD-NMR as a complementary and, in many cases, uniquely informative technique for structure–function analysis in hydrated food systems.

Longitudinal relaxation time (T₁) describes the recovery of magnetization along the magnetic field axis and is governed by rapid local molecular motions in the ns–μs regime (1, 7). Longitudinal relaxation time (T₂) reflects the decay of spin coherence in the transverse plane and is highly sensitive to structural constraints, water–macromolecule interactions, and compartment size (4, 8, 9). Self-diffusion coefficients (D), derived from pulsed field-gradient (PFG) methods, quantify translational mobility and reveal pore geometry, tortuosity, and mesoscale confinement (8, 10, 11). Together, these parameters capture a continuum of molecular-to-macroscopic mobility within hydrated food systems (9).

Importantly, the relaxation and diffusion principles described above do not represent isolated physical observables but form a hierarchical continuum linking molecular motion, mesoscale confinement, and macroscopic food functionality. In the following section, these TD-NMR parameters are explicitly mapped onto water-holding capacity (WHC), texture, and microstructural organization, illustrating how multiscale NMR signatures translate into performance-relevant food properties.

To unify these signatures, we apply a three-tier multiscale model that links molecular dynamics, mesoscopic organization, and macroscopic food functionality.

### 1.1 Tier 1: molecular dynamics (ns–μs)

Fast rotational and segmental motions of water molecules, lipids, and protein segments govern dipole–dipole relaxation, expressed most directly in T₂ values (1, 7). Increased molecular mobility results in longer T₂, whereas restricted environments—such as protein aggregation, cross-linking, or fat crystallization—reduce mobility and shorten T₂ (8, 9).

### 1.2 Tier 2: mesoscopic organization (μm)

Micrometer-scale compartments—including muscle fibers, pores, fat droplets, and protein–polysaccharide networks—determine the distribution of T₂ components and influence diffusion behavior (8, 11). These structural constraints generate multicomponent relaxation patterns that distinguish intra- from extra-myofibrillar water in meat (12, 13). Similar principles reveal mesoscale heterogeneity in plant-based matrices (14–17) and emulsion-structured systems (17).

### 1.3 Tier 3: macroscopic functional attributes (mm–cm)

Interactions spanning molecular and mesoscopic scales give rise to macroscopic functional properties such as WHC, texture, juiciness, firmness, and sensory quality (16, 18–21). This tier links TD-NMR parameters to performance-relevant and consumer-perceived attributes (22, 23), enabling non-destructive prediction of food quality.

## 2 Principles of multiscale TD-NMR

### 2.1 Proton relaxation as a signature of water distribution

Water mobility in food systems is strongly influenced by physical constraints imposed by proteins, polysaccharides, and lipids (4, 8, 24). T₂ relaxation distributions provide a sensitive measure of water compartmentalization within complex matrices (13, 25). In muscle systems, distinct T₂ components are commonly assigned to intra-myofibrillar, extra-myofibrillar, and more loosely associated water domains (13). Structural tightening—such as during postmortem pH decline or protein denaturation—reduces water mobility and shortens T₂ values (20, 25), whereas increased hydration or protein unfolding enhances mobility and lengthens T₂ (26).

Plant-based matrices exhibit analogous, composition-dependent patterns governed by protein network formation, polysaccharide interactions, and emulsion organization (15, 16).

T₁ relaxation provides complementary insight by probing fast molecular motions associated with dipolar relaxation (1, 7). Whereas T₂ predominantly reflects compartment size and geometric constraints, T₁ is more sensitive to local rotational freedom and matrix viscosity (2, 3). Increased molecular mobility typically produces longer T₁ values, while network densification, aggregation, or crystallization restrict motion and shorten T₁ (8, 27). Accordingly, T₁ serves as an indicator of protein–water interactions and the effects of thermal or mechanical modification.

### 2.2 Diffusion and microstructural constraints

Self-diffusion coefficients (D), measured by pulsed field-gradient (PFG) TD-NMR, reveal microstructural features such as pore connectivity, tortuosity, and geometric confinement (8, 10). In muscle foods, anisotropic diffusion arises from the alignment of myofibrils and the hierarchical organization of the sarcomere structure (28, 29). In soy-, pea-, or wheat-based gels, reductions in the apparent diffusion coefficient indicate network tightening, pore closure, or reduced water availability within the matrix (14, 15, 30).

T₂–D correlation maps integrate diffusive mobility with structural constraints, enabling the assignment of water populations to specific compartments and interaction states (11, 31, 32).

### 2.3 Multimodal TD-NMR: relaxation, diffusion, exchange, and imaging

Recent advancements allow multiple TD-NMR modalities to be combined within a unified analytical framework:

- *T₁–T₂ correlation:* resolves distinct mobility states and identifies exchange processes between water populations (11, 31, 32).
- *Multiple-quantum (MQ) NMR:* probes molecular ordering, cross-link density, and gel strength in protein- or polysaccharide-based networks (33).
- *Exchange relaxometry:* quantifies water–protein exchange kinetics and evaluates binding dynamics (32).
- *Low-field NMR imaging:* spatially maps moisture distribution and heterogeneity at the millimeter scale (27, 34).

Together, these modalities cover complementary length and time scales, linking molecular motion (sub-μs), mesoscale architecture (1–100 μm), and macroscopic behavior (mm–cm).

## 3 Relationships among WHC, texture, and microstructure

### 3.1 WHC

WHC is strongly linked to water mobility and compartmentalization:

- Shorter T₂ values indicate restricted water mobility and are associated with reduced WHC (13, 19, 20).
- Loss of intra-myofibrillar water is directly linked to cooking loss and structural shrinkage during thermal treatment (26, 28, 35).
- In plant-based gels, densified protein networks and polysaccharide-mediated cross-linking reduce both T₂ and the diffusion coefficient (D), reflecting a decline in WHC (14, 15, 30, 36).

### 3.2 Texture relationships

Relaxation and diffusion parameters correlate with instrumental texture measurements (e.g., texture profile analysis [TPA]):

- Short T₂ values are associated with firmer, more cohesive structures due to reduced water mobility and increased network density (4, 9).
- Long T₂ values correspond to softer, more hydrated matrices with greater molecular mobility and weaker structural constraints (37).

These relationships have been validated across muscle foods, dairy gels, and plant-based analogs (14, 15, 38–40).

### 3.3 Microstructural correspondence

Cryo-SEM, confocal microscopy, and micro-CT support TD-NMR interpretations:

- Myofibrillar shrinkage during heating corresponds to shortened T₂ values (20, 35, 41).
- Emulsion restructuring modifies relaxation distributions by changing the compartmentalization of water and lipids (34).
- Dense soy- or pea-protein gels exhibit reduced diffusion coefficients and stronger confinement, consistent with tighter network organization (14, 15, 30, 42).

Taken together, these findings indicate that TD-NMR provides a quantitative proxy for microstructural organization across diverse food matrices.

## 4 Applications to meat and plant-based foods

### 4.1 Meat systems

Postmortem biochemical changes—including pH decline, proteolysis, and protein denaturation—reshape relaxation populations and diffusion behavior (19, 25, 35). During heating, intra- to extra-myofibrillar water redistribution and progressive water immobilization occur, closely associated with cooking loss and texture transitions (12, 13, 20, 26, 43–45).

### 4.2 Plant-based matrices

Soy-, pea-, wheat-, and mixed-protein systems form networks distinct from animal muscle (14, 15, 46). TD-NMR differentiates hydration states, fat mobility, pore structure, and phase separation, providing mechanistic explanations for differences in firmness, juiciness, and WHC relative to meat (12, 47).

Beyond conventional soy-, pea-, and wheat-based systems, TD-NMR is increasingly relevant for emerging protein formats such as mycoprotein-based foods and hybrid or cultured meat products. Mycoprotein matrices exhibit filamentous fungal networks with distinct water confinement and relaxation behavior compared to globular plant proteins, while cultured or hybrid meat systems combine cellular and extracellular components that generate complex, multicomponent relaxation signatures. TD-NMR provides a non-destructive means to differentiate these architectures and to assess hydration, structural integrity, and processing-induced changes in next-generation protein foods.

### 4.3 Comparative interpretation: meat vs. plant-based burgers

Relaxation–diffusion signatures reveal fundamental structural differences:

- Meat exhibits a hierarchical native protein structure (12, 26, 35).
- Plant-based analogs consist of engineered protein aggregates, emulsions, and composite networks (14, 15, 48, 49).
- Hydration topology, pore geometry, and confinement patterns differ substantially (11).

These differences explain the observed variations in WHC, texture, and cooking performance.

Quantitatively, meat systems typically exhibit narrower and more distinct T₂ populations corresponding to anatomically defined water compartments, whereas plant-based matrices display broader and often overlapping relaxation components reflecting heterogeneous, engineered networks (Table 1). Apparent diffusion coefficients are generally lower in plant-based systems due to tighter confinement within protein–polysaccharide matrices and emulsified fat domains, whereas meat exhibits directionally dependent diffusion arising from aligned myofibrillar structures. These systematic differences provide a quantitative TD-NMR basis for interpreting observed disparities in juiciness, WHC, and thermal behavior.

**Table 1:** Representative TD-NMR signatures in meat and plant-based food matrices.

TD-NMR parameter	Meat systems	Plant-based systems
Dominant T₂ populations	Distinct, well-resolved intra- and extra-myofibrillar water components	Broad, often overlapping relaxation distributions reflecting heterogeneous water environments
Typical T₂ range	Predominantly short to intermediate T₂ values associated with restricted and semi-restricted compartments	Wider and formulation-dependent T₂ range influenced by protein type, hydration level, and matrix design
Diffusion behavior	Anisotropic diffusion aligned with muscle fiber orientation and structural hierarchy	Largely isotropic diffusion governed by polymeric networks and dispersed phases
Structural origin of signal	Native hierarchical muscle architecture (myofibrils, sarcomeres, connective tissue)	Engineered protein–polysaccharide networks and/or emulsion-based structures
Functional implication	Intrinsic WHC linked to juiciness and texture retention	WHC and texture strongly dependent on formulation, processing conditions, and network connectivity

**Figure 1:**
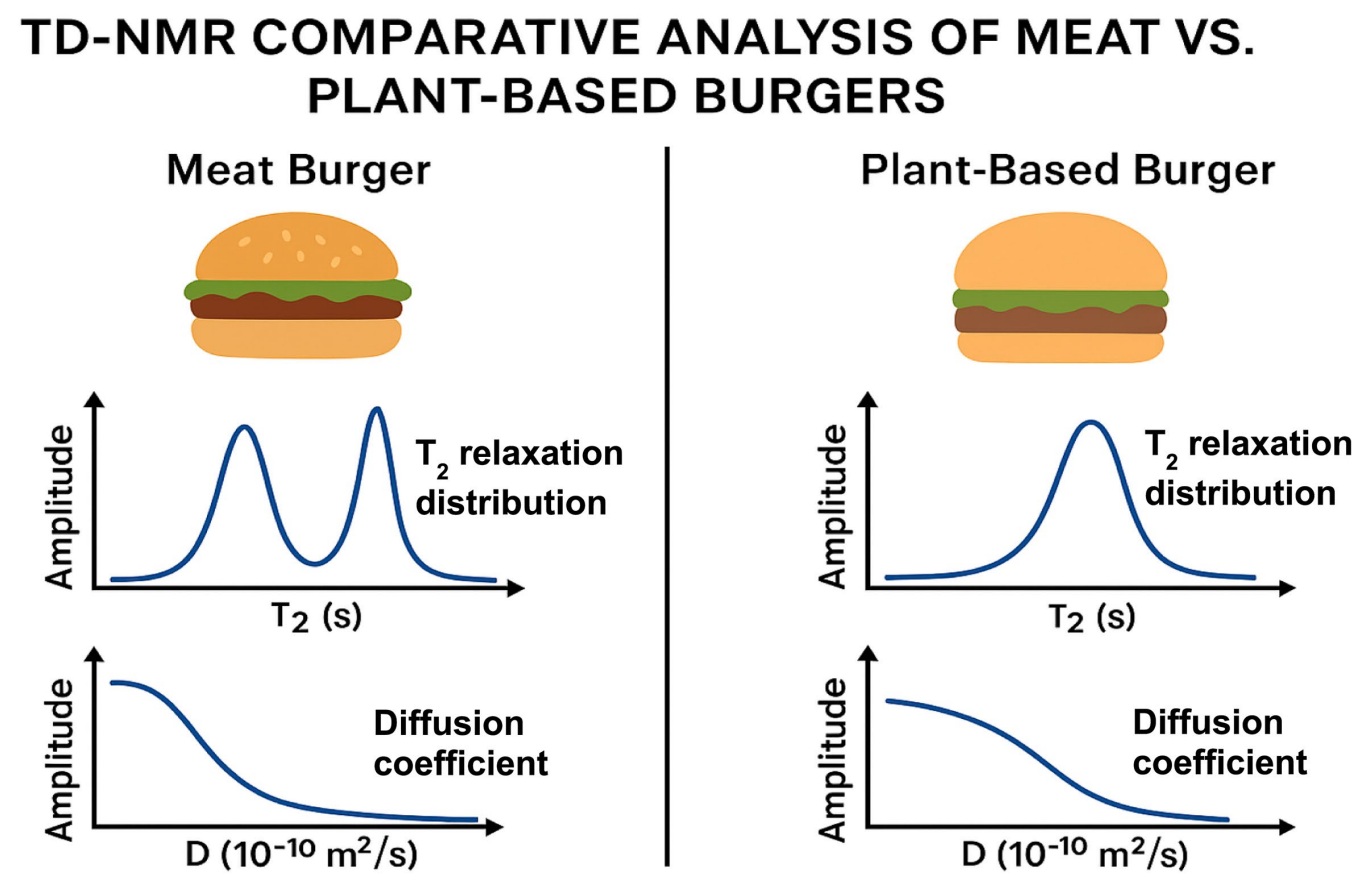
TD-NMR comparative analysis of meat vs. plant-based burgers.

Low-field TD-NMR relaxation and diffusion signatures highlight fundamental differences in water mobility, hydration topology, and microstructural constraints between meat and plant-based burger matrices (Figure 1). Meat exhibits a characteristic multicomponent T₂ relaxation profile with well-resolved populations assigned to intra-myofibrillar, extra-myofibrillar, and loosely bound water domains. This compartmentalization, combined with higher anisotropy and structural ordering, is reflected in its diffusion behavior.

In contrast, plant-based burgers show a broader, more uniform T₂ distribution and a reduction in apparent diffusion coefficient, consistent with engineered protein aggregates, emulsified fat domains, and composite gel networks lacking the hierarchical organization of muscle. These mobility signatures provide a mechanistic basis for differences in juiciness, WHC, and texture between the two product types.

## 5 Toward a unified multiscale TD-NMR framework

The integrated framework encompasses:

1. *Molecular mobility (T₁, T₂):* Relaxation times reflect proton mobility and binding states at the molecular level—for example, water associated with proteins, lipid protons, or more mobile water in bulk/free states (50, 51).
2. *Mesoscale constraints (diffusion, exchange):* Diffusion-weighted and correlation TD-NMR (e.g., D–T₂, T₁–T₂) reveal compartmentalization (pores, fat globules, protein networks), exchange kinetics, and confinement effects—bridging molecular mobility with meso−/microstructural organization (52, 53).
3. *Macrofunctional traits (texture, WHC, juiciness):* TD-NMR mobility and distribution parameters correlate with large-scale, functional food-quality metrics, including WHC, drip/cook loss, juiciness, texture, and stability during storage or processing (12, 47, 54).
4. *Practical perspective/applications:* TD-NMR parameters can be leveraged for real-time process monitoring, non-destructive quality assessment, and optimization of formulation variables. Multicomponent relaxation and diffusion metrics have been successfully applied to track structural transitions and predict product performance under industrial processing conditions (55–58). These approaches enable rapid, non-invasive evaluation of water distribution, protein–matrix interactions, and texture-related properties, supporting improved process control and consistent product quality.

This materials-science–inspired multiscale logic—linking molecular mobility, confinement, and macroscopic function—is increasingly recognized as a robust, non-destructive framework for probing water–fat–matrix interactions across scales in food systems (55, 57–62).

## 6 Future perspectives

### 6.1 Machine learning and chemometric analysis

Recent studies demonstrate the value of combining low-field NMR with chemometric and machine learning (ML) tools to extract meaningful quality metrics from complex food matrices (63, 64). For example, a convolutional neural network (CNN) applied to T₂ relaxation data from a low-field sensor classified edible oils by oxidation level (non-oxidized, partially, highly oxidized) with ~95% accuracy, illustrating how ML can robustly map raw decay data to functional quality labels (65). As food systems become increasingly complex (multi-component, multiscale), ML and chemometric pipelines are expected to become essential for handling high-dimensional datasets, which may include relaxation, diffusion, exchange, imaging, and metadata.

### 6.2 Physics-guided and Bayesian inversion methods

Traditional inversion methods (e.g., Laplace inversion for T₂ distributions) are limited, particularly with overlapping or broad components in complex food matrices. Incorporating physical constraints—such as diffusion–relaxation coupling or exchange models—into Bayesian or regularized inversion approaches can yield more accurate and reproducible distributions of relaxation and diffusion parameters. This approach addresses ambiguity in interpreting NMR data and better links NMR-derived parameters to real structural or compositional features, meeting the growing demand for quantitative, comparable metrics across food types.

### 6.3 Integration with rheological and sensory models

NMR-based analysis has shown that water/fat mobility, compartmentalization, and distribution are closely linked to macroscopic properties such as texture, WHC, and stability (50). The next step is formalizing these links via multivariate or mechanistic models that relate NMR observables (relaxation times, diffusion coefficients, compartment sizes) to rheological or sensory outcomes (e.g., juiciness, tenderness, mouthfeel). Predictive models could transform NMR from a descriptive to a predictive tool, supporting process control during mixing, cooking, and storage, as well as product development for texture and formulation design.

### 6.4 Inline low-field TD-NMR sensors for industrial monitoring

Low-field, single-sided, and benchtop NMR systems offer rapid, non-destructive, minimal-preparation assessments suitable for industrial environments (55). For instance, combining a low-field H^1^ LF-NMR relaxation sensor with ML classification enabled real-time monitoring of oil oxidation levels in industrial settings (64). As NMR hardware continues to decrease in size, cost, and complexity (e.g., benchtop permanent magnets, surface probes), embedding NMR-based sensors into production lines could allow continuous monitoring of water content, fat distribution, moisture migration, oxidation, and structural integrity.

### 6.5 Reference databases for standardization and benchmarking

The development of curated reference databases of TD-NMR signatures represents a critical step toward standardization and broader adoption of NMR-based food analysis. Such databases could be maintained through collaborative efforts involving academic laboratories, instrument manufacturers, and industrial stakeholders. Essential metadata would include sample composition, processing history, temperature, magnetic field strength, pulse sequence parameters, inversion methods, and normalization protocols. Well-curated databases would enable benchmarking across products and laboratories, support calibration transfer and machine-learning model generalization, and facilitate regulatory and industrial acceptance of TD-NMR as a quantitative quality-control tool.

**Figure 2:**
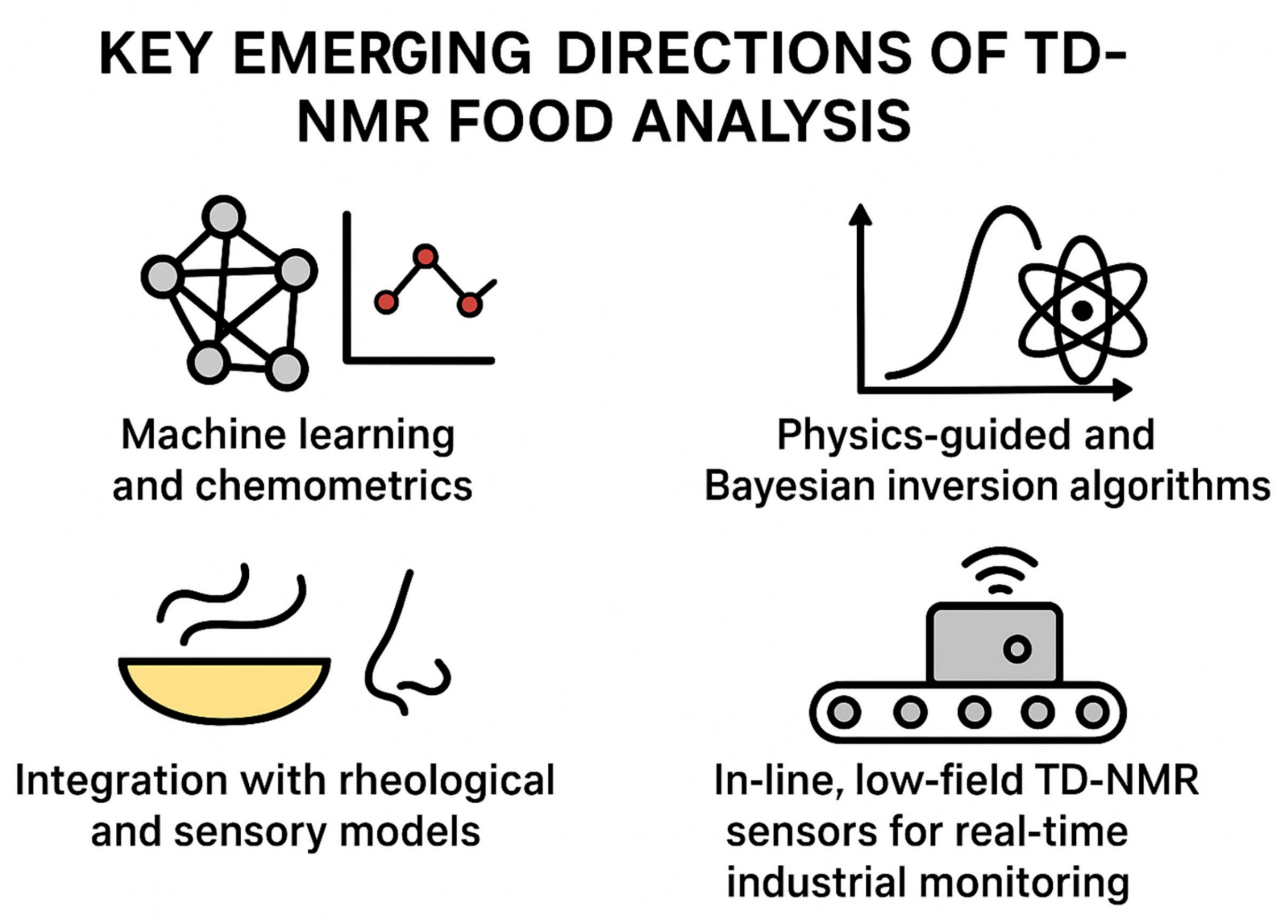
Key emerging directions in TD-NMR food analysis.

Overview of key technological and analytical advancements shaping next-generation TD-NMR applications in food science (Figure 2). Machine learning and chemometrics enable the extraction of higher-order patterns from multidimensional relaxation–diffusion datasets, while physics-guided and Bayesian inversion methods enhance the accuracy and robustness of relaxation distributions. Integration with rheological, microstructural, and sensory models is advancing TD-NMR toward fully predictive quality frameworks. Improvements in low-field instrumentation are driving the development of inline, real-time industrial sensors, and emerging community databases are establishing standardized mobility benchmarks across diverse food systems.

## 7 Conclusion

TD-NMR offers comprehensive, multiscale insights into water distribution, molecular mobility, and microstructural organization in both meat and plant-based foods. Over time, the technique has evolved into a robust analytical framework with strong mechanistic foundations. With the growing adoption of advanced processing technologies, alternative protein sources, and data-driven modeling approaches, TD-NMR is poised to remain a central tool in food research, quality assessment, and product development.
